# Enhancing growth and physiological traits in alfalfa by alleviating salt stress through biochar, hydrogel, and biofertilizer applications

**DOI:** 10.3389/fmicb.2025.1560762

**Published:** 2025-05-14

**Authors:** Dilfuza Jabborova, Yu Zhang, Saleh S. Alhewairini, Zafarjon Jabbarov, Jayanthi Barasarathi, Tokhtasin Abdrakhmanov, Otamurod Imomov, Sokhibjon Abdusamatov, Baljeet Singh Saharan, Riyaz Sayyed

**Affiliations:** ^1^Institute of Genetics and Plant Experimental Biology, Uzbekistan Academy of Sciences, Kibray, Uzbekistan; ^2^Key Laboratory of Tropical Fruit Biology, Ministry of Agriculture and Rural Affairs/Key Laboratory of Hainan Province for Postharvest Physiology and Technology of Tropical Horticultural Products, South Subtropical Crops Research Institute, Chinese Academy of Tropical Agricultural Sciences, Zhanjiang, China; ^3^Department of Plant Protection, College of Agriculture and Food, Qassim University, Buraidah, Saudi Arabia; ^4^Faculty of Biology, National University of Uzbekistan, Tashkent, Uzbekistan; ^5^Faculty of Health and Life Sciences (FHLS), INTI International University, Nilai, Malaysia; ^6^Department of Microbiology, Chaudhary Charan Singh Haryana Agricultural University, Hisar, India; ^7^Department of Biological Sciences and Chemistry, College of Arts and Science, University of Nizwa, Nizwa, Oman

**Keywords:** biofertilizer, nitrogen fixation, plant growth promotion, salinity stress, sustainable agriculture

## Abstract

**Introduction:**

Salinity is a significant abiotic stress that affects the growth, physiology, and yield of crop plants across the globe. Sustainable measures to mitigate saline soil and prevent yield losses require immediate attention. The present study aimed to determine the impacts of hydrogel, biochar, and biofertilizer on alfalfa growth and physiological properties under salt stress.

**Methods:**

The experiment was performed in a randomized block design with three replications on the dried bottom of the Aral Sea, consisting of control (T1), hydrogel alone (T2), biochar alone (T3), and biofertilizer alone (T4). Plant growth parameters, root morphological traits, and physiological properties were analyzed after 60 days of sowing.

**Results:**

The results showed significant improvement in shoot length, shoot dry weight, and root dry weight in biochar alone (T3) and biofertilizer alone (T4) treated plants compared to control (T1) and hydrogel (T2). However, the application of biochar alone (T3) exhibited more pronounced effects compared to other treatments.

**Discussion:**

Biochar treatment resulted in the highest chlorophyll a and total chlorophyll contents under salt stress. Soil amendments with biochar, hydrogel, and biofertilizer promote alfalfa growth and yield and help mitigate the adverse impact of salt stress.

## 1 Introduction

Biochar (BC), a carbon-rich residue, has received considerable attention (Elkhlifi et al., 2023). Due to its large specific surface area, it has a unique porous structure with excellent biochemical stability and a high adsorption capacity for sorbing and releasing mineral nutrients (Kul, 2022). It is a rich source of mineral nutrients, including carbon, nitrogen, and sulfur. Biochar is used as a soil amendment to improve soil health, sequester atmospheric carbon, and provide energy (Abbas et al., 2020; Mansoor et al., 2021). Additionally, applying BC to soil results in long-term carbon sequestration (Sun et al., 2020). Through improved nitrogen fixation, soil biochar supplementation improves the supply of essential cations and mineral nutrients, including phosphorus and total nitrogen, boosting soil and water fertility. Under salinity stress, biochar promotes plant growth and soil properties (Su et al., 2024) and increases crop yield. Additionally, it reduces environmental contamination (Singh et al., 2009). Plant growth and productivity can be increased by adding biochar directly or indirectly. It influences plant growth directly by enhancing nutrient uptake and indirectly by improving the physicochemical and biological composition of the soil (Aubertin et al., 2021). The amount of mineral nutrients in the soil, including K^+^, Ca^2+^, Mg^2+^, and P, is increased using biochar as a nutrition provider (Aubertin et al., 2021). Biochar may also impact soil nutrients by modifying microbial and fungal metabolism and diversity, affecting the availability and quality of soil nutrients (Lehmann et al., 2011). Most of the carbon in biochar remains in the soil, making it an appealing alternative to mineral fertilizers for soil amendment (Schmidt et al., 2011; Abel et al., 2013; Ekinci and Turan, 2021; Laird et al., 2017).

The Papilionoideae subfamily includes the perennial forage legume alfalfa (*Medicago sativa* L) (Acharya et al., 2020; Acharya et al., 2020; Yazıcılar et al., 2021; Al-Turki et al., 2023). Due to its wide range of adaptability, high yield, excellent quality, and tolerance to repeated cuttings, lucerne is a significant feed source for cattle businesses worldwide (Acharya et al., 2020; Ghani et al., 2022). It can produce seeds, pasture, hay, silage, dehydrated goods, and improved soil (Acharya et al., 2020; Ghani et al., 2022; Al-Turki et al., 2023). The legume alfalfa is somewhat tolerant to salinity (Al-Turki et al., 2023; Yang et al., 2023). Using traditional breeding techniques, various lucerne cultivars with enhanced salt tolerance have been created (Shrivastava and Kumar, 2015). However, salt tolerance in alfalfa is difficult to genetically increase since numerous genes regulate salt tolerance and include a variety of biochemical and physiological pathways, which makes alfalfa plants’ physiological and genetic response to salt stress complex (Al-Turki et al., 2023; Shrivastava and Kumar, 2015).

One of the most critical stresses restricting agricultural production is the salt in the soil (Al-Turki et al., 2023). Saline soil has excessive soluble salts (such as chloride, sulfate, sodium, calcium, magnesium, and potassium carbonates) in the root zone (Shrivastava and Kumar, 2015). This soil type makes it difficult for plants to get water and nutrients from the soil and can harm the plants (Shrivastava and Kumar, 2015; FAO, 2022). Saline soil has an electrical conductivity (EC) of more than four dS m^−1^ saturation extract in the root zone at 25°C and 15% exchangeable sodium (Wiebe et al., 2007). Long recognized as a widespread environmental occurrence, salinization is now recognized as a global problem of land degradation (Timm et al., 2018; Rolfe et al., 2019; Al-Turki et al., 2023), with a higher frequency in arid and semi-arid areas. In the world, salinity (397 million hectares) and its associated sodicity (434 million hectares) damage more than 6% of the total land area (FAO, 2022).

For instance, salinization has impacted the agricultural productivity of 6 million ha of agricultural land in the Canadian Prairies (FAO, 2022; Al-Turki et al., 2023) and more than 10 million hectares of agricultural land in the North American Great Plains (Wiebe et al., 2007; Al-Turki et al., 2023; Khan et al., 2025). The Food and Agriculture Organization (FAO) has calculated that 0.25–0.50 million ha of irrigated areas become unfit for cultivation yearly due to salt buildup over time (Rolfe et al., 2019). One of the best methods for ensuring sustainable crop production is creating crop cultivars resistant to soil salt (Al-Turki et al., 2023).

Numerous bacteria in the rhizosphere are closely related to plants and perform host services that fluctuate in response to stressors and environmental stimuli (Bright et al., 2025; Ferioun et al., 2025; Ben-Laouane et al., 2020; Kapadia et al., 2021; Khumairah et al., 2022). Pioneering research, for example, suggests that plants use the “cry-for-help” technique by altering the chemical composition of their root exudates to attract helpful bacteria to help them overcome abiotic and biotic challenges (Anli et al., 2020). Arbuscular mycorrhizal fungi (AMF) 31, 32 and rhizobia 33 are two examples of helpful soil microorganisms that can be used in sustainable agriculture programs to combat salt problems (Afrouz et al., 2023; Sagar et al., 2020; Boutasknit et al., 2020; Jabborova et al., 2022; Sagar et al., 2022a; Sagar et al., 2022b). It is known that both mycorrhizal and rhizobial symbioses boost the host plants’ ability to withstand abiotic challenges by influencing various pathways and enhancing their growth and productivity (Vafa et al., 2021). Compared to a single-strain inoculation, it has been suggested that co-inoculation and co-culture of microorganisms may be more advantageous and promote plant growth (Boutasknit et al., 2020). AMF increases photosynthetic rate, enhances nutrition and water intake, and activates the antioxidant system to reduce ROS damage (Jat and Ahlawat, 2006). Glomalin, a glycoprotein molecule that increases soil aggregate stability and water potential, is the primary mechanism by which AMF could improve soil physicochemical and biological qualities (Garg et al., 2019). Legumes and Rhizobium species work together to fix atmospheric N2, providing agriculture with a regenerative nitrogen source (Boutasknit et al., 2020). Due to the growth-promoting characteristics of these microorganisms, which include phytohormone and biofilm generation, P and K solubilization, N fixation, particular enzymatic activity secretion, and P and K solubilization, plant growth and biomass can be enhanced (Boutasknit et al., 2020; Khan et al., 2021; Kusale et al., 2021; Kapadia et al., 2022). The present study evaluated the effects of biochar, hydrogel, and biofertilizer on growth, physiological traits of alfalfa, and soil properties under saline soil.

## 2 Materials and methods

### 2.1 Biochar, soil, and seed

The municipal solid waste biochar was obtained from the Soil Sciences Department of Biology faculty, NUU. Pyrolysis of municipal solid waste biochar was carried out at 500°C for 40 min. The municipal solid waste biochar traits are shown in Table 1. Alfalfa seeds were provided by the Scientific Research Institute of Plant Genetic Resources, National Center for Knowledge and Innovation in Agriculture, Uzbekistan. Tables 2, 3 show analyses of the sands’ physicochemical properties and salinity levels.

**Table 1:** Municipal solid waste biochar characteristics.

Biochar	BOC (g/kg)	BOM (g/kg)	TN (g/kg)	TP (g/kg)	TK (g/kg)	AN (mg/kg)	AP (mg/kg)	AK (mg/kg)	pH
Mean contents	330.6	570	0.26	4.48	42.6	237.8	0.77	688.9	8.01

Sand samples were collected from the dry bottom of the southern part of the Aral Sea.

BOC, biochar organic carbon; BOM, biochar organic matter; TN, total nitrogen; TP, total phosphorus; TK, total potassium; AN, available nitrogen; AP, available phosphorus; AK, available potassium.

**Table 2:** Physicochemical properties of sands in the dried bottom of the Aral Sea.

Sand	SOCg/kg	SOMg/kg	TN g/kg	TP g/kg	TK g/kg	AN mg/kg	AP mg/kg	AK mg/kg	pH
Mean contents	0.87	1.53	0.52	0.23	0.71	1.24	1.13	42.95	8.5

SOC, soil organic carbon; SOM, soil organic matter.

**Table 3:** Salinity level of the sands in the dried bottom of the Aral Sea.

Sand	НСО_3_ (g/kg)	CI (g/kg)	SO_4_ (g/kg)	Ca (g/kg)	Mg (g/kg)	Na (mg/kg)	K (mg/kg)
Mean contents	0.02	4.24	12.43	2.12	1.48	632.22	3.24

Alfalfa (*Medicago sativa* L.) seeds were used for field experiments.

### 2.2 Experimental design

Experimental work was conducted on the dried bottom of the Aral Sea to study the effect of biochar, hydrogel, and biofertilizer on alfalfa growth, root morphology, and physiological traits. It was carried out in a randomized block design with three replication experiments. Experimental treatments included biochar, hydrogel, and biofertilizer [Yer malxami (*Azotobacter chroococcum*)]. After 60 days, plants were harvested, and plant height, shoot, and root dry weights were measured.

### 2.3 Measurement of root morphological traits of alfalfa

The roots were carefully washed with water. The whole root system was spread out and analyzed using a scanning system (Expression 4990, Epson, CA) with a blue board as a background. Digital images of the root system were analyzed using Win RHIZO software (Régent Instruments, Québec, Canada). The total root length, the root surface area, the root volume, the projected area, and the root diameter were evaluated.

### 2.4 Physiological parameter measurement

Physiological parameters, total chlorophyll, chlorophyll a, chlorophyll b, and carotenoid contents in alfalfa, were measured by Hiscox and Israelstam (1979). A fresh leaf sample (50 mg) of alfalfa was cut, and dimethylsulfoxide (5 mL) was added to test tubes. The test tubes were incubated at 28°C for 4 h. The absorbance of the extract was taken at 470 nm, 645 nm, and 663 nm using a spectrophotometer against a DMSO blank. The chlorophyll a (Chl a), chlorophyll b (Chl b), total chlorophyll, and carotenoid contents were determined using the following equation:


$$\text{Chlorophyll}\;a\;(\mathrm{mg}/g)=12.7\;(A663)-2.69\;(A645)x\frac{V}{W}$$

$$\text{Chlorophyll}\;b\;(\mathrm{mg}/g)=22.9.7\;(A645)-4.68\;(A663)x\frac{V}{W}$$

$$\text{Total chlorophyll}\;(\mathrm{mg}/g)=20.2\;(A645)+8.02\;(A663)x\frac{V}{W}$$

$$\begin{array}{l} \text{Carotenoids}\;(\mathrm{mg}/g)=\\ \;\;\;\;\;\;\;[(1000\;x\;A470)–(3.27\;x\;\mathrm{Chl}\;a+104\;x\;\mathrm{Chl}\;b)]x\frac{V}{W} \end{array}$$

The relative water content of leaves in alfalfa was analyzed using Barrs and Weatherley’s method (Barrs and Weatherley, 1962). A volume of 100 mg of expanded fresh leaf sample (FW) was placed immediately after sampling in Petri plates filled with double-distilled water for 4 h at 28°C. The samples were then taken out, and a blotted dry and turgid weight (TW) was recorded. After that, the samples were kept in an oven at 70°C overnight, and the dry weight (DW) was recorded.

### 2.5 Analysis of soil nutrients

The agrochemical parameters of the soil were analyzed after cultivation. The humus content was analyzed using Tyurin’s modified method. The soil’s P, K, and total N contents were analyzed using the technique (GOST 26107-84, GOST 26107-84).

### 2.6 Analysis of soil enzymes

Soil enzyme (Invertase and catalase) activities were also assayed using the method by Ngun et al. (2020). An invertase activity of dried soil (5.0 g) was used in the experiment. Then, the dried soil and sucrose solution with water were added. After quantification, the glucose content was measured using a spectrophotometer. For catalase activity, soil (2.0 g) was mixed with H_2_O_2_ (5.0 mL) and double-distilled water (40.0 mL). The catalase activity was then determined using a spectrophotometer.

### 2.7 Statically data analysis

ANOVA was used to examine experimental data with IBM SPSS Statistics 20. Analysis of variance (ANOVA) was conducted to compare the significant or insignificant difference in the effect of treatments on growth, root morphological, and physiological properties of alfalfa using Duncan’s multiple range tests with the least significant difference at a 5% significance level (α = 0.05).

## 3 Results

### 3.1 Morphological traits of Alfalfa

Treatment of biochar (T3) in saline sand significantly increased the shoot length (cm) by 33.83 ± 1.66, followed by biofertilizer (30.23 ± 1.69), hydrogel (17.24 ± 1.15), and the control, which is 11.85 ± 0.98. The maximum shoot dry weight (g) was observed in plants treated with biofertilizer (7.38 ± 0.02), followed by biochar treatment (6.38 ± 0.03) and hydrogel treatment (5.54 ± 0.02), compared to control. Hydrogel treatment resulted in the highest root dry weight (0.98 ± 0.01) among all treatments and the control. For biochar treatment, the root dry weight, i.e., 0.94 ± 0.01, was compared to biofertilizer and control. The root dry weight was 0.85 ± 0.02 for biofertilizer treatment compared to control. The root dry weight of saline soil without any treatment was 0.65 ± 0.04 (Table 4).

**Table 4:** Plant growth as affected by biochar, hydrogel, and biofertilizer in saline sand.

Treatments	Shoot length (cm)	Shoot dry weight (g)	Root dry weight (g)
Control	11.85 ± 0.98a	4.08 ± 0.05a	0.65 ± 0.04a
Hydrogel	17.24 ± 1.15b	5.54 ± 0.02b	0.98 ± 0.01d
Biochar	33.83 ± 1.66d	6.38 ± 0.03c	0.94 ± 0.01c
Biofertilizer	30.23 ± 1.69c	7.38 ± 0.02d	0.85 ± 0.02b

Data are means of three replicates with standard error. ± = standard deviation. The same letters denote statistically insignificant differences, while treatments with different letters significantly differ in the values (*p* < 0.05).

### 3.2 Root morphological traits of Alfalfa

Biochar treatment enhanced the total root length, root projection area, root surface area, root volume, and root diameter by 46.53 ± 1.90 cm, 4.66 ± 0.22 cm^2^, 8.29 ± 0.31 cm^2^, 0.37 ± 0.01 cm^3,^ and 1.86 ± 0.05 mm, respectively, compared to hydrogel, biofertilizer, and control. There is no significant difference between hydrogel and biofertilizer; both enhance the root parameters compared to the control. The hydrogel treatment results in a root diameter of 1.60 ± 0.10 cm, a root volume of 0.33 ± 0.01 cm^3^, a root surface area of 8.07 ± 0.08 cm^2^, a root projected area of 4.20 ± 0.10 cm, and a total root length of 39.60 ± 0.70 cm. The biofertilizer treatment improved the total root length, root projection area, root surface area, root volume, and root diameter by 37.53 ± 0.67 cm, 3.90 ± 0.10 cm^2^, 8.29 ± 0.31, 0.37 ± 0.01 cm^2^ and 1.86 ± 0.05 mm compared to the control. In the saline soil without any treatment, the total root length, root projection area, root surface area, root volume, and root diameter were 31.60 ± 2.86 cm, 3.50 ± 0.10 cm^2^, 7.63 ± 0.86 cm^2^, 0.26 ± 0.02 cm^3^, and 1.09 ± 0.17 mm, respectively (Table 5).

**Table 5:** Root parameters of alfalfa as affected by hydrogel, biochar, and biofertilizer in saline sand.

Treatments	Total root length (cm)	Root projected area (cm^2^)	Root surface area (cm^2^)	Root volume (cm^3^)	Root diameter (mm)
Control	31.60 ± 2.86a	3.50 ± 0.10a	7.63 ± 0.86a	0.26 ± 0.02a	1.09 ± 0.17a
Hydrogel	39.60 ± 0.70b	4.20 ± 0.10c	8.07 ± 0.08a	0.33 ± 0.01b	0.98 ± 0.01d
Biochar	46.53 ± 1.90c	4.66 ± 0.22d	8.29 ± 0.31a	0.37 ± 0.01c	1.86 ± 0.05c
Biofertilizer	37.53 ± 0.67b	3.90 ± 0.10b	7.92 ± 0.16a	0.28 ± 0.03a	1.45 ± 0.15b

Data are means of three replicates with standard error. ± = standard deviation. The same letters denote statistically insignificant differences, while treatments with different letters significantly differ in the values (*p* < 0.05).

### 3.3 Physiological properties of Alfalfa

**Figure 1:**
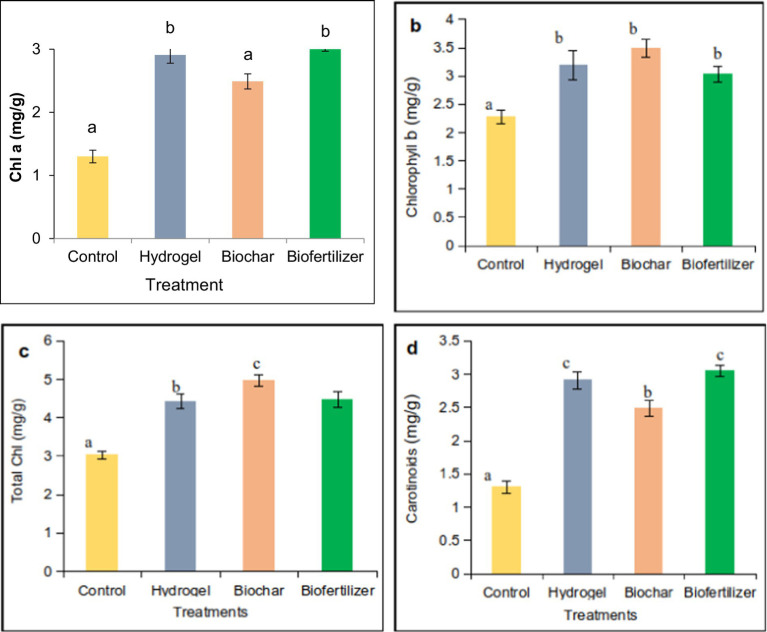
Photosynthetic pigments of alfalfa are affected by hydrogel, biochar, and biofertilizer in saline sand. Chlorophyll a **(a)**, chlorophyll b **(b)**, total chlorophyll **(c)**, and carotenoids **(d)**.

Biochar treatment enhanced the chlorophyll a content compared to the control (Figure 1). Treatment with hydrogel also increased the chlorophyll content compared to the control. Considerable differences were observed between biochar, biofertilizer, treatment with hydrogel, and control. The control has a minimum chlorophyll content.

Hydrogel and biochar were achieved in the content, and biofertilizer with the maximum chlorophyll b content was added to the control. There was no significant difference between biochar and biofertilizer. Total chlorophyll was maximum in the biochar treatment compared to hydrogel, biofertilizer, and the control.

Treatments of hydrogel and biofertilizer also have higher chlorophyll content than the control. There is no significant difference between hydrogel and biofertilizer. Hydrogel and biofertilizer treatments have a high carotenoid content compared to biochar and control. There was no significant difference between hydrogel and biofertilizer. Biochar treatment also has higher carotenoid contents than the control (Figure 1).

**Figure 2:**
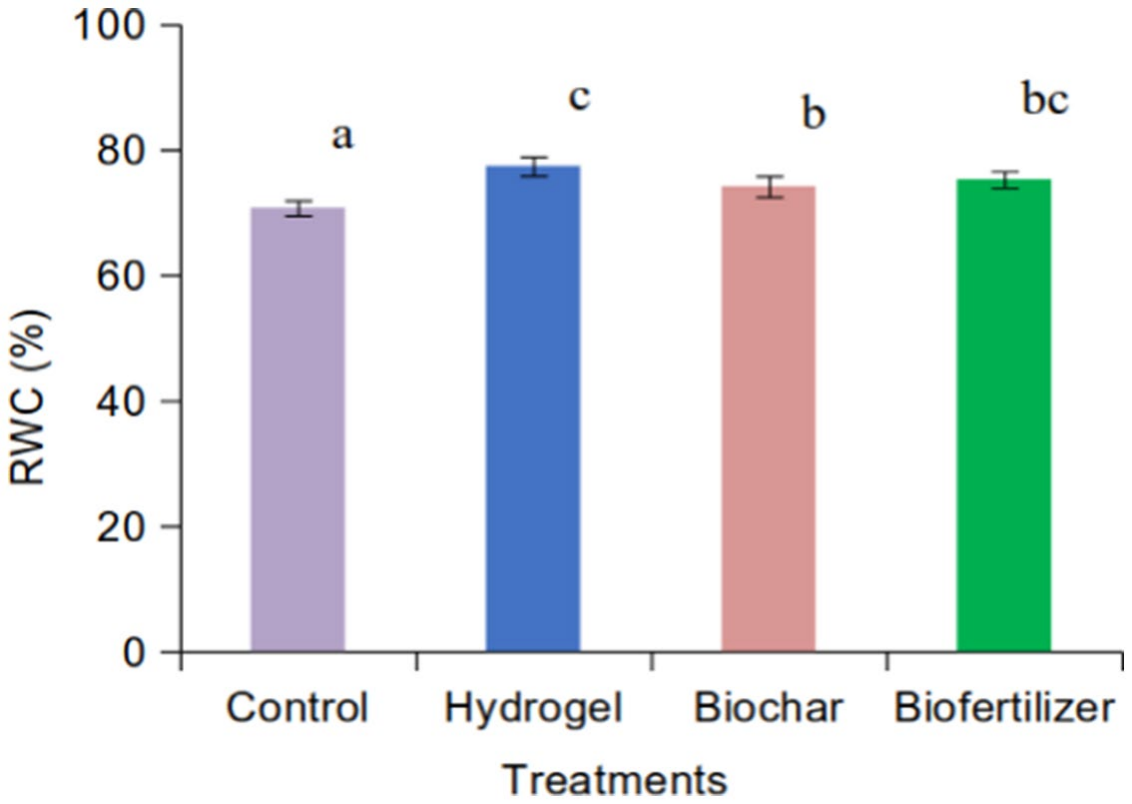
Relative water content of alfalfa as affected by hydrogel, biochar, and biofertilizer in saline sand.

The relative water content (%) increases when treated with hydrogel compared to saline sand’s biofertilizer, biochar, and control. There is no significant difference between hydrogel and biofertilizer. Biochar treatment also increases the RWC compared to control. There is no significant difference between biochar and biofertilizer (Figure 2).

### 3.4 Plant and soil nutrients

Plant nutritional value increases when treated with hydrogel, biochar, and biofertilizer compared to the control (Table 6). Maximum N (%) is increased when treated with hydrogel, biochar (1.86 ± 0.01%), and biofertilizer (1.68 ± 0.01%) compared to biochar and control. There is no significant difference between hydrogel and biofertilizer. Compared to the control, treatment with biochar enhances the total N %, i.e., 1.65 ± 0.03%. In control, the total N % is 1.16 ± 0.01%. Biochar treatment increased the total P (%) by 0.94 ± 0.01% compared to hydrogel, biofertilizer, and control. Hydrogel treatment enhances total P % to 0.87 ± 0.01% compared to biofertilizer and control. Biofertilizer treatment increases the total P% to 0.72 ± 0.01% compared to control. Total K% increased when treated with biochar, i.e., 2.15 ± 0.04, compared to hydrogel, biofertilizer, and control. Hydrogel treatment increases the total K % to 2.02 ± 0.01% compared to biofertilizer and control. Total K % increase in untreated saline soil, i.e., 1.96 ± 0.01, compared to biofertilizer. Biofertilizer treatment total K % 1.72 ± 0.01.

**Table 6:** Plant nutrients of alfalfa as affected by hydrogel, biochar, and biofertilizer in saline sand.

Treatments	Total N (%)	Total P (%)	Total K (%)
Control	1.16 ± 0.01a	0.69 ± 0.01a	1.96 ± 0.01b
Hydrogel	1.86 ± 0.01c	0.87 ± 0.01c	2.02 ± 0.01c
Biochar	1.65 ± 0.03b	0.94 ± 0.01d	2.15 ± 0.04d
Biofertilizer	1.68 ± 0.01c	0.72 ± 0.01b	1.72 ± 0.01a

Data are means of three replicates with standard error. ± = standard deviation. The same letters denote statistically insignificant differences, while treatments with different letters significantly differ in the values (*p* < 0.05).

The soil nutrients of alfalfa are affected by hydrogel, biochar, and biofertilizer in saline sand (Table 7). Biochar, hydrogel, and biofertilizer increase the total N%, i.e., 0.042 ± 0.001, 0.040 ± 0.001, and 0.040 ± 0.001, compared to control. There is no significant difference between hydrogel, biochar, and biofertilizer. Biochar treatment increases the total P% by 0.029 ± 0.001% compared to hydrogel, biofertilizer, and control. There is no significant difference between hydrogel and biofertilizer. In biofertilizer treatment, the total K% was 0.029 ± 0.001%, and the hydrogel total K% was 0.026 ± 0.001% compared to control. In untreated soil, total K%, i.e., 0.022 ± 0.002%. Humus % enhanced by all three treatments, hydrogel (0.163 ± 0.006%), biofertilizer (0.157 ± 0.006%), and biochar (0.025 ± 0.001%) compared to control. There is no significant difference between hydrogel, biochar, and biofertilizer. In control, the humus % is 0.157 ± 0.006%.

**Table 7:** Soil nutrients of alfalfa as affected by hydrogel, biochar, and biofertilizer in saline sand.

Treatments	Total N (%)	Total P (%)	Total K (%)	Humus (%)
Control	0.017 ± 0.001a	0.020 ± 0.001a	0.022 ± 0.002a	0.157 ± 0.006a
Hydrogel	0.040 ± 0.001b	0.024 ± 0.001b	0.026 ± 0.001b	0.163 ± 0.006b
Biochar	0.042 ± 0.001b	0.029 ± 0.001c	0.122 ± 0.003c	0.025 ± 0.001b
Biofertilizer	0.040 ± 0.001b	0.023 ± 0.001b	0.029 ± 0.001b	0.157 ± 0.006b

Data are means of three replicates with standard error. ± = standard deviation. The same letters denote statistically insignificant differences, while treatments with different letters significantly differ in the values (*p* < 0.05).

### 3.5 Soil enzyme activities

The catalase activity is enhanced by biofertilizer treatment up to 15.47 ± 0.03% compared to hydrogel, biochar, and control. Through biochar treatment, the activity of catalase, i.e., 9.43 ± 0.03%, was compared to hydrogel and control. Hydrogel treatment enhances the catalase activity to 8.82 ± 0.06% compared to the control. The control showed the activity of catalase to 2.83 ± 0.04%. Invertase activity is significant to hydrogel and biochar treatment, i.e., 3.52 ± 0.02% compared to biofertilizer and control. Biofertilizer treatment enhances invertase activity to 3.20 ± 0.02% compared to the control. The control activity of invertase to 3.02 ± 0.03% is shown in Table 8.

**Table 8:** Enzyme activities of soil as affected by hydrogel, biochar, and biofertilizer in saline sand.

Treatments	Catalase activity (mL KMnO_4_ g ^−1^ soil h^−1^)	Invertase activity (μg glucose·g^−1^soil·h^−1^)
Control	2.83 ± 0.04a	3.02 ± 0.03a
Hydrogel	8.82 ± 0.06b	3.52 ± 0.02c
Biochar	9.43 ± 0.03c	3.52 ± 0.02c
Biofertilizer	15.47 ± 0.03d	3.20 ± 0.02b

Data are means of three replicates with standard error. ± = standard deviation. The same letters denote statistically insignificant differences, while treatments with different letters significantly differ in the values (*p* < 0.05).

## 4 Discussion

Seed germination and plant growth can be inhibited or delayed by salt stress (Dash and Panda, 2001; Jabborova et al., 2023a; Jahan et al., 2023). Salinity stress can harm plants at any stage of growth, but the plant’s early establishment significantly impacts the output (Al-Turki et al., 2023; Sagar et al., 2022a). Previous studies have demonstrated the negative impacts of salt stress on plant growth parameters (Guo et al., 2007; Singh et al., 2009; Ur Rahman et al., 2021). Numerous studies have shown that plant growth is one of the most critical agricultural indicators of salt stress tolerance.

Normal root and shoot growth are significantly inhibited by salinity stress. The plant’s performance is frequently impacted by the roots’ direct exposure to salinity (Römheld and Kirkby, 2010). Degl’Innocenti et al. (2009) state that potassium is crucial for conveying photosynthates for root growth. Due to osmotic pressure and decreased water intake, the fresh weight of roots may drop. High electrical conductivity also decreases hydraulic conductance (Meguekam et al., 2021). A potential buildup of high salt uptake decreased potassium uptake and decreased dry matter content, reducing root dry weight (Meguekam et al., 2021). Plant development was decreased due to the roots’ exposure to excessive salinity and potassium deficit (El-Iklil et al., 2000; Turan et al., 2007).

Salt-induced weakening of the protein–pigment–lipid complex or elevated chlorophyllase enzyme activity were the causes of this effect (Hand et al., 2017). Due to its detrimental effects on membrane stability, decreased chlorophyll concentration under salt stress is a frequently documented phenomenon in research (Ashraf and Harris, 2013). Plants sensitive to salt have a lower chlorophyll content (Stepien and Johnson, 2009; Mukarram et al., 2022). Mukarram et al. (2022) found that chlorophyll fluorescence, chlorophyll content, and plant development were all reduced by high salt concentrations (240 mM). Several studies demonstrate that salt stress lowers chlorophyll content (Lee and Van Iersel, 2008; Garcia et al., 2019). Maintaining an acceptable level of relative water content is a salt stress tolerance criterion as it affects leaves’ metabolic activity and survival (Sun et al., 2020). Under salt stress, RWC likewise decreased, and water deficit increased in the roots and leaves of Iris lacteal seedlings (Hniličková et al., 2017). While RWC did not begin to decline until 200 mM NaCl in the salt-tolerant, the osmotic potential did with rising NaCl concentrations. The high salt content in the rhizosphere affects the root’s ability to absorb water efficiently, lowering RWC and the soil’s water potential (Römheld and Kirkby, 2010). Salinity causes the plant to accumulate excess ions and lose water, which reduces the osmotic potential.

Numerous studies have demonstrated that biochar helps plants overcome saline stress and grow well under saline soil conditions (Sultan et al., 2024; Murtaza et al., 2024; Duan et al., 2024). Hydrogel improved plant growth under salt stress (Pettinelli et al., 2024; Soliman et al., 2024).

Applying biochar, hydrogel, and biofertilizers is a sustainable remedy to mitigate soil salinity and improve plant growth (Al-Turki et al., 2023; Jabborova et al., 2022).

Jabborova et al. (2020, 2022) have reported the positive impacts of the co-inoculation of biochar and mycorrhizae on growth and nutrient enrichment in soybeans. Kapadia et al. (2022) reported the plant growth-promoting and salinity-ameliorating effects of halophilic bacteria on various horticulture crop plants under greenhouse and field studies. They found that applying halophilic bacteria improves antioxidant machinery that helps overcome salts’ harmful effects. In another study, Kapadia et al. (2021) reported the positive impact of the application of a consortium of PGPR on salinity mitigation, growth promotion in tomato plants, and enrichment of soil nutrients.

The effect of biochar alone has been widely studied, but the impact of biochar in combination with PGPR has been explored less. Gul et al. (2023) examined the effect of biochar and PGPR on barley. This combination significantly improved barley’s growth, physiology, and biochemical contents compared to control. The combination improved chlorophyll content, antioxidant enzymes, and the physicochemical properties of the soil. They suggested adding biochar and PGPR to enhance plants’ soil fertility, crop productivity, and antioxidant defense systems.

Many studies have claimed the positive effects of the combination of biochar and PGPR on soil quality and plant growth promotion under normal and stressed conditions and found that both of these additives complement each other (Kanwal et al., 2017). Various studies reported a significant increase in crop yield in co-applied treatments compared to single application (Malik et al., 2022). Yan et al. (2024) reported significant improvement in the alfalfa dry weight in leaves, stalks, and roots following biochar application. It also enhanced the chlorophyll content and soil nutrient availability, particularly the accessibility of carbon, nitrogen, and phosphorus. The treatment lowered soil pH and enriched microbial diversity. They recommended a moderate application of biochar (10–20 t/ha) to improve the Alfa-Alfa growth and soil health, offering a practical approach to the sustainable farming of alfalfa. Jabborova et al. (2023b) reported the positive effects of biochar treatment on plant growth and physiological traits of alfalfa under salinity stress. They found biochar to be an effective way to mitigate salt stress in crops, reduce salt levels in the soil, improve soil structure, and increase the bioavailability of essential nutrients, enhancing crop growth and yields.

*Azotobacter* sp. inoculation offers broader, legume, non-specific positive effects on the rhizosphere of a wide range of crops. *Azotobacter* sp. improves plant biomass (Holatko et al., 2024). Holatko et al. (2024) reported that *Azotobacter* sp., in combination with *Rhizobium*, enhances plant height by 15%, grain yield by 113%, root nodules by 50%, and photosynthetic pigments by 240% over the control.

Sun et al. (2025) reported that the inoculation of PGPR into alfalfa can improve the plant’s tolerance to salinity stress. They found that the inoculation with PGPR in alfalfa decreased the soil pH and electrical conductivity and increased the soil’s nitrogen, potassium, phosphorus, and organic matter concentrations. They claimed that inoculation with PGPR reduced soil salinity and improved nutrient contents in the rhizosphere, supporting more plant growth under salinity stress conditions.

## 5 Conclusion

In summary, this is the first time to study the effects of biochar, hydrogel, and biofertilizer on alfalfa growth, root morphological traits, and physiological properties under saline sand. Biochar, hydrogel, and biofertilizer improved alfalfa plant growth, root morphological features, physiological properties, and soil enzymatic activity. Thus, biochar application could benefit alfalfa growth and physiological properties and improve soil fertility.

## Data Availability

The original contributions presented in the study are included in the article/Supplementary material, further inquiries can be directed to the corresponding authors.
